# Trends, patterns and determinants of long-acting reversible methods of contraception among women in sub-Saharan Africa

**DOI:** 10.1371/journal.pone.0217574

**Published:** 2019-06-04

**Authors:** Sunday A. Adedini, Olusola Akintoye Omisakin, Oluwaseyi Dolapo Somefun

**Affiliations:** 1 Medical Research Council, Respiratory and Meningeal Pathogens Research Unit, University of the Witwatersrand, Johannesburg, South Africa; 2 Programme in Demography and Population Studies, Schools of Public Health and Social Sciences, University of the Witwatersrand, Johannesburg, South Africa; 3 Demography and Social Statistics Department, Federal University, Birnin-Kebbi, Nigeria; Anglia Ruskin University, UNITED KINGDOM

## Abstract

**Background:**

Method-specific contraceptive prevalence varies widely globally, as huge variations exist in the use of different types of contraception, with short-term methods being the most common methods in sub-Saharan Africa (SSA). Evidence is scanty on the trends, patterns and determinants of long-acting reversible contraceptive (LARC) methods in SSA. This study aimed to address this knowledge gap.

**Methods:**

Using a pseudo longitudinal research design and descriptive and inferential statistics, we analysed Demographic and Health Survey data of eight countries selected on the basis of contraceptive prevalence rates across SSA. Multinomial logistic regression modelling was used to tease out the predictors of the uptake of LARC methods in the selected countries.

**Results:**

Findings exhibit a steady but slow upward trend in LARC methods across selected countries, as a marginal increase was recorded in LARC uptake over a 10-year period in many of the selected countries. Results established significant predictors of LARC methods uptake, including fertility-related characteristics, age, level of education, work status, wealth index and exposure to mass media. This study underscored the need to address various barriers to the uptake of LARC methods in SSA. It is recommended that governments at different levels undertake to cover the costs of LARC methods in order to increase access and uptake.

## Introduction

Rapid population growth remains a major concern in many sub-Saharan African countries (SSA). This is due to its implication for diverse socio-economic ills and developmental and health challenges, including poor maternal and neonatal health, poor capital investment, environmental degradation, and poverty[1]. Africa’s population has continued to increase much faster compared to other regions of the world, and the population figure is projected to account for more than half of world’s total population by 2050 [2]. Fertility rate varies significantly across countries in the SSA, with total fertility rate (TFR) ranging from 2.9 in Botswana to 7.2 in Niger [2]. One major reason for the persistent high fertility level and high maternal and child mortality rate across many SSA countries is the low level of contraceptive uptake and high unmet needs for contraception, either for limiting or spacing [3–5]. Although, contraceptive prevalence rate (CPR) has increased across a number of SSA countries (such as Malawi, South Africa, and Rwanda), the region still has one of the lowest rates of CPR globally [6] with a huge sub-regional differences [7].

A critical look at the sub-regional disparities in contraceptive prevalence across SSA reveals an interesting pattern. For instance, Southern African sub-region has a high contraceptive prevalence rate of about 62 percent, almost exclusive of modern methods and unmet need for family planning is relatively low at 13 percent [8]. This is in contrast to Western Africa where the unmet need is 25 percent and contraceptive prevalence rate is estimated at 15 percent [9]. As expected, these averages vary within the geographic regions. For example, modern contraceptive prevalence rate (mCPR) was estimated at 11 percent among Nigerian women, 18 percent among women in Ghana and 9 percent among their counterparts in Senegal [10]. These low rates of mCPR have been associated with high rates of unintended pregnancy in the region [11]. These have serious public health implications as unintended pregnancies are a leading cause of maternal and child mortality in SSA [12].

Although the annual number of global maternal mortality has substantially decreased from 532000 in 1990, to 303000 in 2015, it still remains very high in sub-Saharan Africa, as the region accounts for around two-thirds of the current level of maternal deaths (66.3%) [13]. The use of appropriate contraceptives among women which varies at different phases in their lives has been identified as a critical component in their reproductive health and socio-economic development. Very importantly, family planning has been reported to be key in the reduction of poverty, improved economic growth, increased female productivity by reducing their fertility and ensuring child survival and improved maternal health [14, 15]. It has also been suggested that continued investment in family planning could be crucial for the attainment of the sustainable development goals [16, 17].

A number of interventions have been put in place to improve contraceptive use among women but the socio-economic differences in the use of contraception still persists [18]. These disparities arise as a result of disadvantaged women being deprived of contraceptives they want to use to protect them from unwanted pregnancies [19]. According to Creanga, Gillespie [20], examining differences in contraceptive use and fertility intentions through an equity lens allows us to understand whether women are being deprived of something they wish they had (i.e. right types of family planning) to avoid something they do not desire (i.e. pregnancy). With respects to family planning types, studies have established that women who intend to stop childbearing but use a short-term or spacing method of contraception are not meeting their need, and neither are women who merely want to space births but use a long-term or permanent contraceptive method [5, 21].

Specifically, contraceptive methods can be grouped into two categories. These are long-acting reversible or permanent contraceptive methods (like intrauterine devices, implants, and sterilization) and short-term methods (like pills, condoms, spermicides, injectable, and other modern methods, and all traditional methods). Long-acting reversible or permanent contraceptive methods are generally used to limit childbearing, whereas short acting methods are important for birth delay and birth spacing. The factors associated with the use of these methods varied [22]. Long acting contraceptives have been described to have low failure rate, safer and cost effective compared to short acting contraceptives [23, 24]

It has been established that women in sub-Saharan Africa are often unable to obtain or use modern contraception, particularly the long acting methods, for many reasons associated with both supply and demand sides[25–27]. They rely primarily on traditional and short-acting contraception, which are prone to incorrect or inconsistent use and failure [28, 29]. Meanwhile, many long-acting reversible contraceptive (LARC) methods provide more than 10 years of highly effective protection against pregnancy.

LARC methods are among the safest, most cost-effective, and reliable forms of contraception available today [30, 31]. Despite this, LARC methods are still being underutilized in SSA, where they could benefit millions of women seeking to control their fertility. Relatively few studies have sought to examine the determinants of these methods. It is important to have a summarized and up-to-date evidence on the trends, patterns and determinants of LARC uptake as this would deepen understanding about the challenges facing intending users of these methods of contraception. Besides, evidence from this analysis could guide the design of appropriate programmes and strategies to increase the availability, accessibility and utilization of LARC across the SSA. Therefore, a study on the uptake of long acting contraceptive methods is a top priority action to improve its accessibility and utilization. Against this background, this study aimed to document the trends (increase or decrease) and patterns of long-acting reversible contraceptive methods and identify its determinants in the selected countries.

## Data and methods

### Study design and participants

The study undertook secondary analysis of Demographic and Health Survey (DHS) data which were obtained using cross-sectional study design. The study utilized data from eight different countries, representing the four sub-regions in SSA namely: Malawi and Rwanda in Eastern Africa, Cameroon and Chad in Central Africa, Zambia and Zimbabwe in Southern Africa and Ghana and Mali in Western Africa. These representative countries were selected on the basis of their contraceptive prevalence rates and subject to the criterion of having conducted the most recent Demographic and Health Survey (DHS) since 2010. We selected the most recent Demographic and Health Survey data and two other data points for earlier years for each of the selected countries in order to present trend analysis. In terms of inclusion or selection criteria, generally the selected countries have relatively higher CPR than many other countries in the region and this is a necessary condition for selecting these country in order to have plausible findings on LARC use which is mainly low in sub-Saharan Africa. The selected countries are heterogeneous, hence we controlled for country as a variable in our analysis.

Data were obtained from the DHS website, with permission from the DHS Program. These include information on socio-economic, bio-demographic characteristics, and contraceptive practices among women of reproductive ages (15–49 years). We use three standard DHS datasets for each of the eight selected countries. These are data from 2004, 2010 and 2016 Malawi DHS; 2005, 2010 and 2015 Rwanda DHS; 1998, 2004 and 2011 Cameroon DHS; 1997, 2004 and 2015 Chad DHS; 2002, 2007 and 2014 Zambia DHS; 2006, 2011 and 2015 Zimbabwe DHS; 2003, 2008 and 2014 Ghana DHS; and 2001, 2006 and 2013 Mali DHS.

Data were pooled into a single file for all the selected countries. As shown in Table 1, the total sample size from the pooled data analysed in this study was 257,998. The study employed a pseudo longitudinal research design in that it examines the trends, patterns and determinants of LARC uptake over time in the selected countries.

**Table 1:** Survey year and analytic samples for selected countries.

Country	Data point/survey year	Weighted samples
Chad	2004, 2007, 2015	7454, 6085, 17719
Cameroon	1998, 2004, 2011	5501, 10656, 15426
Ghana	2003, 2008, 2014	5691, 4916, 9396
Malawi	2004, 2010, 2016	11698, 23020, 24562
Mali	2001, 2006, 2013	12849, 14583, 10424
Rwanda	2005, 2010, 2015	11321, 11671, 13497
Zambia	2002, 2007, 2014	7658, 7146, 16411
Zimbabwe	2006, 2011, 2015	8907, 9171, 9955
**Total**		**257,998**

### Variables measurement

The dependent variable is the use of LARC method, and was treated as a multinomial measure with three categories, which are (i) ‘not using any method’, coded as ‘0’; (ii) ‘using LARC’ (including the IUD and Implants/Norplant), coded as ‘1’; and (iii) ‘using other methods’ (all of which are different from the LARC such as pill, injections, condom, withdrawal, periodic abstinence and so on), coded as ‘2’. ‘Not using any method’ is the base outcome and this means that *mlogit* syntax in Stata software reports coefficients for the effect of selected explanatory variables on ‘the use of LARC methods’ and ‘uptake of short-acting method’ relative to the base category (i.e. ‘not using any method’). Hence, the results present if any independent variable significantly influences the likelihood of using ‘LARC method’ or ‘short-acting method’, with ‘not using any method’ as the reference category. The multinomial logistic regression modelling was undertaken to tease out the odds of using LARC method compared to the other two categories. The selected independent variables (and their respective categories) include respondent’s current age (15–24, 25–34 and 35+), level of education (no formal education, primary, secondary and higher), occupation (unemployed, managerial, clerical/ agric. and labour), wealth index (poorest, poorer, middle, richer and richest) and exposure to mass media (no exposure at all and having an exposure). Other selected covariates are place of residence (urban and rural), country of residence (Cameroon, Chad, Ghana, Malawi, Mali, Rwanda, Zambia and Zimbabwe), marital status (never married, married/ living with partner, widowed, separated and divorced); fertility factors namely CEB (none, 1–4 children and 5 and above) and desire for more children (want another and no desire).

### Statistical analysis

Data were analysed with Stata software (version 13.0). Multiple bar chart was drawn which visually displays the trends and patterns in the use of LARC across the selected countries. The bivariate analysis was carried out using Chi-square test in order to measure the significance of association between the predictor variables and use of LARC from the pooled data for all the selected countries. In addition to identifying the determinants of LARC, we also present the patterns or differentials in LARC uptake to show differences in level of use of LARC between women with low socio-economic characteristics and those with high socio-economic characteristics. Further, at the multivariable level of analysis, we predicted the influence of the individual characteristics and fertility-related variables on the relative risk of using LARC by employing the multinomial logistic regression model from the pooled data for all the selected countries.

## Results

### Trends and differentials in LARC uptake by selected characteristics

**Fig 1:**
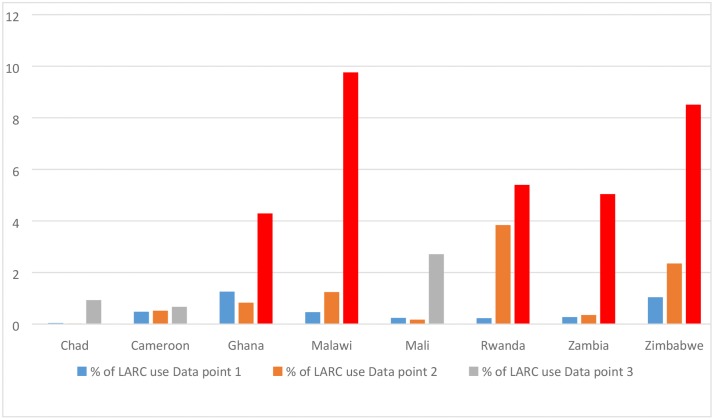
Trend in LARC use in selected SSA countries.

There has been a general upward trend showing that LARC uptake has overtime continued to gain some increase among users in most sub-regions in SSA (as shown in Fig 1). For instance in Malawi, LARC uptake increased from 0.46% in 2004 to 9.76% in 2016 and in Zimbabwe, LARC uptake steadily increased from 1.04% in 2006 to 8.51% in 2015.

**Fig 2:**
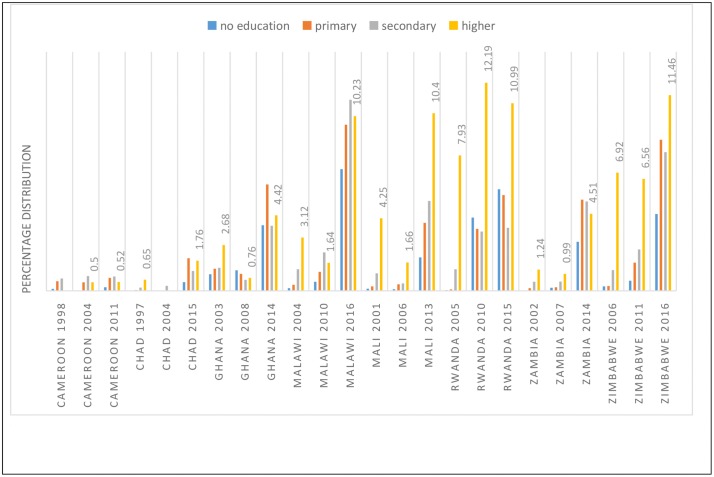
Percentage distribution of the use of LARC in selected SSA countries by level of education.

Differentials in the use of LARC were associated with the level of education, wealth quintile and place of residence. Education was positively associated with the use of LARC particularly among countries in Eastern and Southern Africa (see Fig 2). In Malawi, for instance, the use of LARC among women who attained higher education had increased from 3.12% in 2004 to 10.23% in 2016. Likewise in Zimbabwe, the use of LARC among women who had attained higher education had increased from 6.92%% in 2006 to 11.46% in 2016.

**Fig 3:**
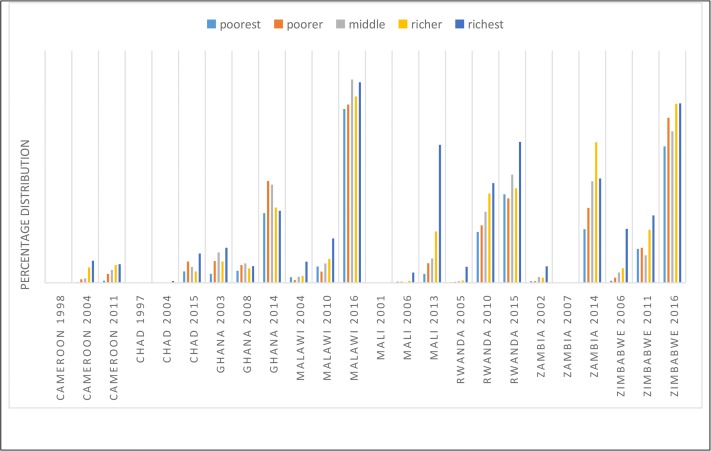
Percentage distribution of the use of LARC in selected SSA countries by wealth quintile.

LARC uptake varied by wealth quintile–particularly among selected countries in Eastern and Southern Africa (Fig 3). Rwanda’s use of LARC among women from richest households had risen from 0.83% in 2005 to 7.28% in 2015. Similar trend occurred in Zimbabwe where use of LARC among women from poorest households (0.1% in 2006) and those from richest households (2.79% in 2006) had risen to 7.05% and 9.27%, respectively in 2016.

**Fig 4:**
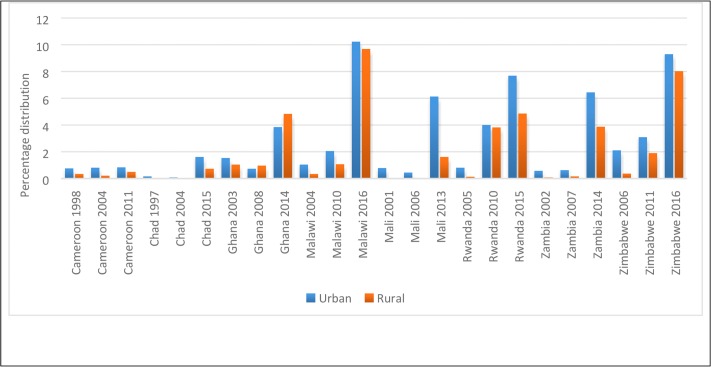
Percentage distribution of the use of LARC in selected SSA countries by place of residence.

With regards to the place of residence, greater proportions of women in the urban areas than those in the rural areas adopted LARC particularly among selected countries in Eastern and Southern Africa (as shown in Fig 4). In Malawi, for instance, the use of LARC in urban areas (1.04% in 2004) and rural areas (0.33% in 2004) had increased to 10.21% and 9.66% respectively in 2016, while use of LARC in urban and rural Rwanda had increased from 0.79% and 0.11% in 2005 to 7.68% and 4.85% respectively in 2015.

### Association between LARC and Socioeconomic and demographic/Fertility characteristics in selected SSA countries

Table 2 presents results from bivariate analysis which explored the relationship between the use of LARC method and selected characteristics. As shown in the table, some of the characteristics are significantly associated with LARC uptake. For instance, the younger age group 15–24 had lower proportion of use of LARC (1.34%) compared to the older age groups 25–34 and 35+ (4.03% and 2.88% respectively). Just 1.0% of women who had no education were using LARC, compared to 5.3% among those with higher education. With regard to marital status, LARC varied among the respondents who were separated (3.97%), divorced (3.72%), married/ living with partner (3.29%), widowed (1.64%) and never married (0.52%).

**Table 2:** Percentage distribution of women using LARC in selected sub-Saharan Africa countries by Sociodemographic/Fertility characteristics.

Characteristics	Not using any method (%)	Using LARC (%)	Using other methods (%)	Chi-square
**Age**				
15–24	82.91	1.34	15.75	1145.3***
25–34	66.6	4.03	29.36	
35+	71.24	2.88	25.88	
Total	74.57	2.61	22.82	
**Education**				
No education	88.49	1	10.51	947.4***
Primary	70.12	3.13	26.75	
Secondary	67.73	3.32	28.95	
Higher	58.58	5.3	36.12	
Total	74.57	2.61	22.82	
**Marital status**				
Never married	90.67	0.52	8.814	1030.2***
Married/ living with partner	68.06	3.29	28.66	
Widowed	85.4	1.64	12.97	
Separated	76.73	3.97	19.3	
Divorced	75.64	3.72	20.65	
Total	74.57	2.61	22.82	
**Occupation**				
Unemployed	79.23	1.89	18.88	211.9***
Managerial	56.49	6.26	37.25	
Clerical/ Agric.	72.52	2.8	24.69	
Labour	72.6	3	24.41	
Total	74.57	2.61	22.82	
**Wealth Index**				
Poorest	78.22	2.2	19.57	78.3***
Poorer	74.8	2.48	22.73	
Middle	73.47	2.84	23.69	
Richer	70.24	3.06	26.7	
Richest	69.41	3.84	26.75	
Total	72.96	2.94	24.1	
**Mass media exposure**				
No exposure	79.62	2.31	18.07	385.0***
At least one exposure	71.17	3.29	25.54	
Total	73.85	2.98	23.17	
**Place of residence**				
Urban	70.82	2.98	26.2	103.3***
Rural	76.28	2.44	21.28	
Total	74.57	2.61	22.82	
**Country**				
Cameroon	75.46	0.59	23.96	545.5***
Chad	94.29	0.54	5.17	
Ghana	78.65	2.58	18.77	
Malawi	62.14	4.62	33.24	
Mali	91.51	0.89	7.6	
Rwanda	76.19	3.33	20.49	
Zambia	68.63	2.8	28.57	
Zimbabwe	56.48	4.12	39.4	
Total	74.57	2.61	22.82	
**Total children ever born**				
None	93.11	0.11	6.78	1682.1***
1–4 children	66.55	3.78	29.67	
5 and above	71.02	2.93	26.05	
Total	74.57	2.61	22.82	
**Desire for more children**				
Want another	78.79	1.84	19.37	1169.3***
No desire	67.76	3.86	28.37	
Total	74.58	2.61	22.81	

***p-value < 0.001

Being employed was associated with LARC use. Respondents in managerial position were the most likely to use the LARC contraceptives (6.26%), followed by those in manual labour (3.0%), clerical or agriculture (2.8%) and the unemployed being the least (1.89%). Increase in wealth index steadily improved the use of LARC, from 2.2% among respondents from poorest households to 3.84% among respondents from richest households. Respondents who had exposure to the mass media may be expected to adopt LARC greater than those who had practically no exposure to mass media. The data showed that 2.31% among the women who had no exposure and 3.29% among the women who had an exposure adopted LARC method.

Furthermore, respondents who were urban-based (2.98%) demonstrated slightly greater use of LARC compared to the rural respondents (2.44%). The distribution of LARC by the selected countries in SSA shows highest proportions of the LARC contraceptive usage in Malawi (4.62%), Zimbabwe (4.12%) and Rwanda (3.33%). The results show highest prevalence of use of LARC among Eastern and Southern African countries. The total number of children ever born was associated with LARC, as 0.11% of women who had no children, compared to 3.78% of women who had 1–4 children and 2.93% of women who had 5 children and above were reported as using LARC contraceptives. Among the respondents, 1.84% of those who wanted more children and 3.86% of those who had no desire for more children adopted the use of LARC.

### Effects of socio-demographic and fertility-related factors on the use of LARC in selected SSA countries

Tables 3 and 4 showed the results of two multinomial logistic regression models. The models compare the uptake of LARC method with the use of short acting method and non-use of any method. Model 1 describes the effects of socio-economic and demographic factors on the use of LARC, while Model 2 describes the effects of the socio-economic and demographic factors on the use of LARC, controlling for the fertility-related factors. In Model 1, changes in the relative risk of LARC were predicted by all the socio-demographic factors with the exception of exposure to mass media. The relative risk of using LARC instead of not using any method was significantly greater in the age group 25–34 (RRR = 2.04; CI: 1.88–2.23; p-value < 0.001) and age group 35+ (RRR = 1.47; CI: 1.33–1.63; p-value < 0.001) compared to the younger age group 15–24. The relative risk of using LARC was more likely to increase consistently by factors of 2.15 among women with primary, 3.19 among women with secondary and 4.65 among women with higher education, than women who had no education (p-value < 0.001).

**Table 3:** Multinomial logistic regression showing the effect of socio-demographic characteristics on the use of contraceptives with ‘not using any method’ as the base outcome.

	Model 1			
	LARC		Other methods	
Characteristics	RRR	95% CI	RRR	95% CI
**Age**				
15–24	1		1	
25–34	2.04	1.88–2.23***	1.51	1.45–1.57***
35+	1.47	1.33–1.63***	1.41	1.36–1.47***
**Education**				
No education	1		1	
Primary	2.15	1.92–2.40***	1.82	1.74–1.91***
Secondary	3.19	2.80–3.63***	2.42	2.29–2.57***
Higher	4.65	3.73–5.79***	3.03	2.70–3.41***
**Marital status**				
Never married	1		1	
Married/ living with partner	11.24	9.69–13.05***	6.38	6.01–6.77***
Widowed	2.72	2.13–3.48***	1.43	1.30–1.58***
Separated	6.83	5.61–8.31***	2.41	2.20–2.64***
Divorced	7.49	6.16–9.09***	2.68	2.45–2.93***
**Occupation**				
Unemployed	1		1	
Managerial	1.35	1.13–1.60**	1.16	1.06–1.27**
Clerical/ agric	1.25	1.13–1.37***	1.25	1.20–1.31***
Labour	1.42	1.30–1.55***	1.3	1.25–1.35***
**Wealth Index**				
Poorest	1		1	
Poorer	1.19	1.07–1.33**	1.14	1.09–1.19***
Middle	1.37	1.22–1.53***	1.16	1.10–1.22***
Richer	1.44	1.28–1.62***	1.3	1.24–1.37***
Richest	1.65	1.44–1.89***	1.23	1.16–1.31***
**Mass media exposure**				
No exposure	1		1	
At least one exposure	1.06	0.98–1.15	1.25	1.21–1.30***
**Place of residence**				
Urban	1		1	
Rural	0.85	0.76–0.96**	0.86	0.82–0.91***
**Country**				
Cameroon	1		1	
Chad	1.39	0.98–1.97	0.24	0.21–0.28***
Ghana	4.26	3.32–5.47***	0.69	0.63–0.76***
Malawi	13.36	10.78–16.54***	1.95	1.80–2.12***
Mali	2.86	2.17–3.77***	0.28	0.25–0.32***
Rwanda	8.77	7.08–10.87***	1.13	1.04–1.23**
Zambia	7.47	5.92–9.43***	1.48	1.36–1.62***
Zimbabwe	10.02	8.03–12.51***	2.31	2.12–2.51***

*p-value < 0.05;

**p-value < 0.01;

***p-value < 0.001

**Table 4:** Multinomial logistic regression showing the effect of socio-demographic and fertility characteristics on the use of contraceptives with ‘not using any method’ as the base outcome.

	Model 2			
	LARC		Other Methods	
Characteristics	RRR	95% CI	RRR	95% CI
**Age**				
15–24	1		1	
25–34	1.29	1.18–1.41***	1.08	1.04–1.12***
35+	0.72	0.64–0.80***	0.85	0.81–0.90***
**Education**				
No education	1		1	
Primary	2.2	1.97–2.46***	1.87	1.78–1.96***
Secondary	3.55	3.11–4.04***	2.65	2.50–2.81***
Higher	6.54	5.16–8.29***	3.93	3.46–4.48***
**Marital status**				
Never married	1		1	
Married/ living with partner	1.93	1.61–2.32***	2.16	2.02–2.30***
Widowed	0.44	0.34–0.58***	0.48	0.43–0.53***
Separated	1.15	0.93–1.43	0.82	0.75–0.91***
Divorced	1.25	1.00–1.55*	0.91	0.82–1.00
**Occupation**				
Unemployed	1		1	
Managerial	1.28	1.07–1.54**	1.12	1.02–1.24*
Clerical/ agric	1.2	1.09–1.32***	1.21	1.16–1.26***
Labour	1.35	1.24–1.48***	1.24	1.20–1.30***
**Wealth Index**				
Poorest	1		1	
Poorer	1.21	1.09–1.35***	1.15	1.10–1.21***
Middle	1.39	1.24–1.55***	1.18	1.12–1.24***
Richer	1.5	1.34–1.69***	1.36	1.29–1.43***
Richest	1.79	1.56–2.05***	1.33	1.25–1.41***
**Mass media exposure**				
No exposure	1		1	
At least one exposure	1.08	1.00–1.17*	1.27	1.22–1.31***
**Place of residence**				
Urban	1		1	
Rural	0.86	0.76–0.96	0.86	0.81–0.91***
**Country**				
Cameroon	1		1	
Chad	1.27	0.89–1.81	0.22	0.19–0.26***
Ghana	4.43	3.44–5.71***	0.71	0.64–0.79***
Malawi	12.42	9.98–15.46***	1.9	1.74–2.07***
Mali	2.92	2.21–3.86***	0.28	0.25–0.32***
Rwanda	8.76	7.04–10.89***	1.15	1.05–1.26**
Zambia	6.61	5.22–8.36***	1.35	1.23–1.47***
Zimbabwe	9.84	7.85–12.34***	2.34	2.15–2.55***
**Total children ever born**				
None	1		1	
1–4 children	41.14	23.63–71.62***	5.88	5.46–6.32***
5 and above	54.33	30.64–96.31***	7.87	7.24–8.54***
**Desire for more children**				
Want another	1		1	
No desire	1.41	1.31–1.52***	1.11	1.08–1.15***

*p-value < 0.05;

**p-value < 0.01;

***p-value < 0.001

In terms of marital status, the relative risk of using LARC was greater by 11.24 times among women who were married or living with a partner, 2.72 times among women who were widowed, 6.83 times among women who were separated and 7.49 times among women who were divorced, compared to women who were never married (p-value < 0.001). Likewise, the relative risk of using LARC was significantly greater by 1.35 times among women in managerial position, 1.25 times among women who were clerical/agric workers and 1.42 times among women who were labour workers, compared to women who were unemployed.

The relative risk of using LARC instead of not using any method was greater by 1.19 times among women from poorer household, 1.37 among women from middle households, 1.44 among women from richer households and 1.65 among women from richest households, compared to the women from poorest households.

As regards the place of residence, the relative risk of using LARC rather than not using any method was significantly lower among rural women when compared to the urban women (RRR = 0.85; CI: 0.76–0.96; p-value < 0.01) and by country, the relative risk of using LARC was significantly greater by 2.86 times in Mali, 4.26 times in Ghana, 7.47 times in Zambia, 8.77 times in Rwanda, 10.02 times in Zimbabwe, 13.36 times in Malawi, compared to Cameroon.

In Model 2, which adjusted for the effects of fertility factors, the socio-demographic factors remained significant predictors of the use of LARC. Likewise, total number of children ever born and desire for more children significantly influenced the relative risk of adopting the LARC method. The relative risk of using LARC was significantly greater among women who had 1–4 children by 41.14 times, and 54.33 times among women who had 5 or more children relative to the women who had no children. Women who had no desire for more children were 1.41 times more likely to adopt the LARC than women who desired to have more children.

## Discussion

The objective of this study was to examine the trends, patters and determinants of long-acting reversible methods of contraception among women in sub-Saharan Africa. Previous studies have established huge variations in method-specific contraceptive prevalence globally, with short-term methods being the most commonly used in SSA [5, 32–34]. Meanwhile, evidence remains sparse on the trend and patterns of LARC use in SSA. Also, factors shaping their uptake across the region are less understood. This study provides empirical evidence on this. Using pseudo longitudinal research design the paper documents interesting findings.

Results from trend analysis demonstrate a steady but sluggish upward slanting in the uptake of LARC methods across countries. This finding lends credence to existing literature regarding the heavy reliance on short-acting contraceptive methods in SSA [28, 29]. Women who intend to stop childbearing but use short-term contraceptives are regarded as having unmet needs [5, 21]. Although our analysis demonstrates a slow-paced increase in the uptake of LARC methods, the observed upward trend suggests increasing availability, accessibility and utilization of the methods across the region.

There were differential patterns in the use of LARC methods by socio-economic characteristics such as educational attainment, wealth status and place of residence. For instance, use of LARC methods was more than three-fold higher among women with higher education compared to those with no education in many of the selected countries. As earlier noted, correct knowledge about LARC methods is a key factor to ensure increased and appropriate use. Similar findings indicate that women from rich households and urban areas had a much higher uptake of LARC methods compared to their poor and rural counterparts. Existing studies have demonstrated generally low level of contraceptive use among poor and rural women–whether short-term modern or traditional methods [33, 35, 36]. Interestingly, the use of LARC method by wealth index showed similar trend for women in poorest households and those in richest households during the same period in Malawi and Zimbabwe. This could possibly be explained by interventions that aim to increase access to long-acting reversible contraceptives in Malawi and Zimbabwe.

Furthermore, our results showed higher uptake of LARC methods among women employed in managerial occupation compared to other categories of women. The plausible reason for this is that women who engage in managerial occupation are likely to be more educated and also afford LARC methods. Besides, the desired or actual fertility level of women who undertake managerial responsibilities is likely to be very low, hence their propensity to adopting long-acting contraceptive method to achieve their fertility preference through appropriate child spacing or limiting. For reasons that appear obvious (such as lack of resources or means), uptake of LARC method was almost non-existent among the unemployed and poor women. A huge body of literature has highlighted high unmet need for contraception among these categories of women [34, 37–39].

As has been previously established [40], uptake of LARC methods was significantly higher in selected East and Southern African countries compared to those in West and Central Africa. Many of the countries in these sub-regions have massive untapped potentials for LARC methods uptake. Thus, findings of this study suggest that programmatic intervention for expansion of LARC methods, particularly across West and Central Africa may be a promising way to support the achievement of the goals of FP 2020 which involve reaching people with unmet needs.

Further, our findings showed that older women were more likely to use LARC methods, compared to their younger counterparts. Possible explanation for this is that older women tend to have more children, have completed their family size and perhaps desire no more children; hence their preference for LARC method compared to short acting contraceptives.

This may seem rational and plausible reason may be because many long-acting contraceptives provide long years of protection against pregnancy [22]. However, LARC methods may also be regarded as suitable contraceptives for younger women, particularly because many of these long-term methods are reversible. Similarly, married women had higher likelihood of using LARC method than those in other categories of marital status. Similar reasons cited above may be advanced for this finding. Specifically, this could be because the desire for more children tends to be lower among married women (who may possibly have completed their family size before opting for LARC method) compared to unmarried women. Single or unmarried women may also adopt appropriate long-term reversible methods, nevertheless fear of side effects such as infertility or delay in getting pregnant may prevent many of them from using LARC methods [41]

Our results also showed that likelihood of using LARC methods was significantly much higher among multiparous women, particularly those who had 5 or more children, compared to their childless counterparts. In addition, women who had no desire for more children were more likely to adopt LARC methods than those who desired more children. This is expected, as long-acting contraceptives are generally viewed as permanent contraceptive methods and are mostly used to limit childbearing [22]. Although LARC methods such as hormonal intrauterine devices (IUDs), copper IUDs, implants, and injections are long-acting, they are completely reversible and can be adopted also as contraceptives for child spacing. Adoption of these methods has faced enormous barriers due to early design errors, problems regarding insertion and removal, as well as myths and misconceptions about side effects [30]. Meanwhile, recent evidence shows that LARC methods that are currently available are safe, easy to use, effective, long lasting, and easily reversible with rapidly restored fertility upon reversal[22, 30].

## Conclusion and recommendation

Although LARC methods have a high cost up-front, in the long term, they are more cost-effective than the short-term methods. This study concludes that the cost of adopting LARC methods may constitute serious demand side barriers for many women in the sub-Saharan Africa, particularly the uneducated, poor and rural women. Thus, this study underscores the need to address various barriers to the uptake of LARC methods in SSA. As part of country-level population policies and programmes aimed at reducing unwanted pregnancies and fertility, it is recommended that governments at different levels undertake to cover the costs of LARC methods in order to increase access and uptake.

## Limitations and strengths of study

This study has some limitations. The temporal sequence of events for some characteristics may present some limitation. Also, being a secondary data analysis, important context- or country-specific characteristics that may influence LARC uptake in different countries could not be explored in the study. This is because such factors were not available in the dataset. Notwithstanding these limitations, this study has filled an important gap in the literature on family planning and reproductive health by providing evidence on the trends, patterns, and determinants of LARC use in sub-Saharan Africa, where the utilization of these methods has remained consistently low for a very long time. This analysis also presents findings that are generalizable and comparable across many sub-Saharan African countries.
